# Systematic Comparison of Atomistic Force Fields for the Mechanical Properties of Double-Stranded DNA

**DOI:** 10.1021/acs.jctc.3c01089

**Published:** 2024-02-27

**Authors:** Carlos Roldán-Piñero, Juan Luengo-Márquez, Salvatore Assenza, Rubén Pérez

**Affiliations:** † Departamento de Física Teórica de la Materia Condensada, Universidad Autónoma de Madrid, E-28049 Madrid, Spain; ‡ Instituto Nicolás Cabrera, Universidad Autónoma de Madrid, E-28049 Madrid, Spain; § Condensed Matter Physics Center (IFIMAC), Universidad Autónoma de Madrid, E-28049 Madrid, Spain

## Abstract

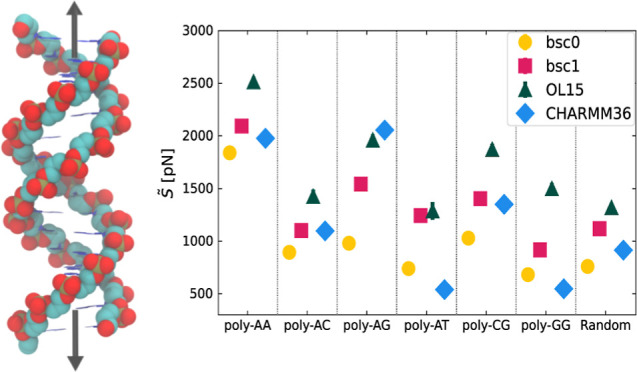

The response of double-stranded DNA to external mechanical stress plays a central role in its interactions with the protein machinery in the cell. Modern atomistic force fields have been shown to provide highly accurate predictions for the fine structural features of the duplex. In contrast, and despite their pivotal function, less attention has been devoted to the accuracy of the prediction of the elastic parameters. Several reports have addressed the flexibility of double-stranded DNA via all-atom molecular dynamics, yet the collected information is insufficient to have a clear understanding of the relative performance of the various force fields. In this work, we fill this gap by performing a systematic study in which several systems, characterized by different sequence contexts, are simulated with the most popular force fields within the AMBER family, bcs1 and OL15, as well as with CHARMM36. Analysis of our results, together with their comparison with previous work focused on bsc0, allows us to unveil the differences in the predicted rigidity between the newest force fields and suggests a roadmap to test their performance against experiments. In the case of the stretch modulus, we reconcile these differences, showing that a single mapping between sequence-dependent conformation and elasticity via the crookedness parameter captures simultaneously the results of all force fields, supporting the key role of crookedness in the mechanical response of double-stranded DNA.

## 1 Introduction

The elasticity of double-stranded DNA (dsDNA) is a key molecular determinant in the many cellular contexts where this molecule is found. For instance, accommodating dsDNA onto the histone core of nucleosomes comes at a significant mechanical cost,^1,2^ which can be alleviated by intrinsic bending of the sequence.^3,4^ Increasing evidence points also to the dsDNA mechanics dependence of transcription factors affinity,^5−8^ thus adding a further layer of complexity to readout mechanisms based on a static description of the system.^5,9−11^ More in general, elasticity affects the DNA response to the mechanical action exerted by proteins and ligands in the most disparate contexts, including genome organization in prokaryotes,^12^ topology regulation,^13^ DNA recombination,^14^ and toxin-induced double-stranded breaks.^15^

This has prompted an intensive research effort in the experimental characterization of the mechanical properties of dsDNA.^4,16−44^ At length scales larger than 10 nm, the mechanics of the duplex is dominated by the persistence length *l*_p_, which regulates the thermally induced bending of the molecule. At standard ionic strength, *l*_p_ lies in the range^4,16−22^ of 45–55 nm. At shorter length scales, the flexibility of dsDNA is also dictated by the stretch modulus *S* and the twist modulus *C*, which account for the deformability of the molecule in elongation and torsion, respectively. These elastic constants are also of interest for large molecules under the presence of mechanical stress, e.g., forces in the range 1–50 pN and torques around 1–30 pN nm. Quantitatively, it has been found that *S* takes typical values within *S* = 900–1600 pN,^20−23^ where the variability might be ascribed to the different techniques employed, particularly optical versus magnetic tweezers,^23^ although a relevant role might be also played by the protocols used for analysis of the force-vs-extension curves. A more uniform range of values has been instead measured for the twist modulus, *C* = 390–460 pN nm^2^.^23−26^ Twist and torsion are also coupled to each other, with dsDNA counterintuitively overwinding upon pulling, as quantified by a negative twist-stretch coupling constant^23,26−28,45,46^ – 120 pN nm < *g* < – 90 pN nm.

All-atom molecular dynamic simulations (AMDSs) are an excellent tool to investigate the mechanical properties of dsDNA, as they enable the inspection of the microscopic mechanisms underlying the global deformation response.^47^ For comparison with experiments, the elongation *L* and the torsion θ of the molecule as a whole are defined starting from atomistic data, and the elastic constants *S*, *C*, and *g* are determined from stress-vs-strain curves^48,49^ or by analyzing the thermal fluctuations of *L* and θ.^50,51^ The parm99 force field^52^ in combination with the bsc0 modification^53^ has been shown to predict elastic constants in good quantitative agreement with the experimental estimations.^46,48,49,54,55^ More recently, further force-field modifications have been proposed—OL15^56^ and bsc1^57^—mainly to improve on the prediction of the helical twist, which was slightly underestimated in bsc0.^53^ A thorough comparison of the predicted structural properties has indicated a similar performance of bsc1 and OL15 in capturing the conformational space of dsDNA.^58^ Due to their excellent structural agreement with experiments and stability over long simulation times, these modifications have now been accepted as the new standards for the simulation of dsDNA. A valid alternative is provided by the CHARMM family of force fields, for which the most up-to-date version—CHARMM36—has been shown to provide solid predictions for structural properties.^59^

The values of *S*, *C*, and *g* predicted by the AMBER and CHARMM force fields have also been found to be in reasonable quantitative agreement with experiments.^50,57,60−62^ For the persistence length, previous works on bsc1 and OL15 have reported either good quantitative agreement^57,60^ or a significant overestimation of *l*_p_,^50^ while to our knowledge, there have been no attempts at estimating *l*_p_ from CHARMM36 simulations. Despite the extensive use of these force fields in the literature, there is a lack of comparative studies highlighting the similarities and differences in the predicted elastic properties within the same sequence context. To our knowledge, only ref (50) computes the values of *S*, *C*, and *g* by employing bsc1 and OL15 for the same dsDNA fragment, while ref (60) performs a comparison of the values of *C* and *l*_p_ obtained for a 32mer via bsc1 and OL15. Another work focused instead on analyzing a random sequence by employing the bsc0 and bsc1 modifications, as well as CHARMM27 and CHARMM36.^62^ Yet, the authors reported a marked instability of the simulations, which prevented computing the elastic constants for CHARMM36.^62^

Future flexibility studies would strongly benefit from a benchmark where the predictions obtained by the various force fields are assessed in similar conditions and in various sequence contexts, as was done previously for the structural features.^58^ To meet this need, here, we present the results of simulations of various dsDNA sequences performed by using either the bsc1 or the OL15 modifications with and without constant pulling forces and compare them with previous reports employing bsc0 on the same set of duplexes.^48,49^ Moreover, we also study the flexibility of the CHARMM36 force field, albeit only in the unperturbed case. The sequences are chosen so as to encompass all the ten distinct base-pair steps.^46,48,49^ We find that the analyzed force fields give similar results for the various elastic constants, although some clear differences are evident. Particularly, the AMBER modifications can be ranked as bsc0 < bsc1 < OL15 according to the predicted value of *S̃*, with bsc0 providing the most flexible values and OL15 corresponding to the stiffest molecules. Even though in all cases the elastic constants were found to be in reasonable agreement with experiments for a random-like sequence, in line with previous literature,^50,57,60,61^ the range of values obtained in the full simulation set and the long-term stability of simulations suggest that bsc1 might be a preferred option for future flexibility studies.

## 2 Methods

### 2.1 Molecular Dynamics

Following previous work focused on the bsc0 force field,^48,49^ we performed AMDSs of the following dsDNA fragments (we write in parentheses the labels that we use in the text to refer to them):

5′-CGCG(AA)_5_CGCG-3′ (poly-AA),

5′-CGCG(AC)_5_CGCG-3′ (poly-AC),

5′-CGCG(AG)_5_CGCG-3′ (poly-AG),

5′-CGCG(AT)_5_CGCG-3′ (poly-AT),

5′-CGCG(CG)_5_CGCG-3′ (poly-CG),

5′-CGCG(GG)_5_CGCG-3′ (poly-GG),

5′-GCGCAATGGAGTACGC-3′ (RNG),

The sequences, named poly-XY, are obtained as pentamers of the step XY, while the sequence RNG contains all possible base-pair step combinations and has been introduced in the literature to mimic the behavior of long random sequences employed in the experiments.^46^ In all cases, the fragment of interest is sandwiched between two handles in order to minimize the end effects.^48^

For each sequence, the dsDNA molecule was built by employing the NAB software within Ambertools19.^63^ The system was then placed in a box of side approximately equal to 9 nm and hydrated with explicit water molecules. In order to ensure overall electrical neutrality, a suitable amount of sodium counterions was added to counterbalance the overall negative charge originating from the phosphate-group moieties. As a control on the relevance of counterion screening, we repeated some simulations by adding 100 mM of NaCl salt. The exact number of ions to be added was determined by following the SLTCAP method^64^ according to the nonlinear reformulation reported in ref (65). For dsDNA, we employed the parm99 force field^52^ with either the bsc1^57^ or the OL15 modifications^56^ as well as the CHARMM36 force field.^59^ Water was modeled according to the TIP3P model,^66^ while the Joung–Cheatham parameters were employed for the sodium counterions.^67^ For some selected cases, we performed simulations also with the OPC model,^68^ to check the robustness of the results with respect to the choice of the water force field (Figure S4 in the Supporting Information). Long-range electrostatic effects were accounted for by employing particle mesh Ewald, while van der Waals interactions were truncated at the real space cutoff (9 Å). We constrained hydrogen-containing bonds by means of the SHAKE algorithm. The integration time step was set to 2 fs.

Starting from the coordinates provided by NAB, the topology and coordinate files for bsc1 and OL15 were obtained via the tleap software within Ambertools19.^63^ In the case of CHARMM36, the NAB coordinates were used as input in the web-based CHARMM-GUI tool^69^ to obtain a topology file in AMBER format.^70^ The topology file was then further edited to set the ion parameters to the Joung–Cheatham values and to introduce the restraining bonds (see below).

**Figure 1:**
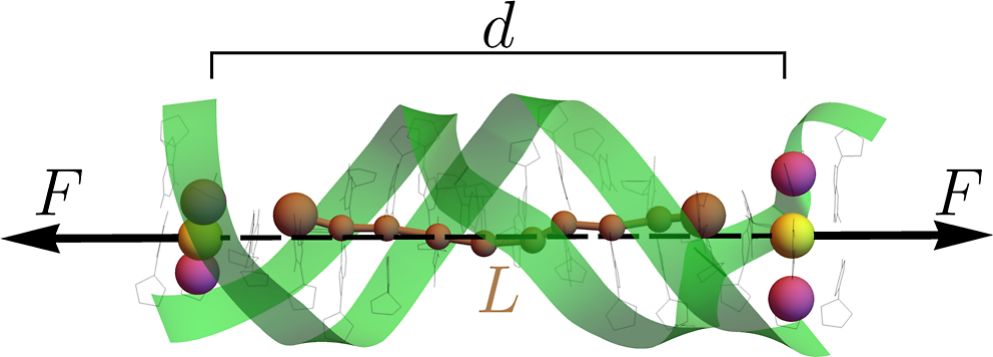
Representative snapshot illustrating the pulling protocol. Two opposite forces of magnitude *F* are applied on the centers of mass (yellow beads) of the C1′ atoms (magenta beads) of the second and second-to-last base pairs, separated by a distance *d*. The forces are aligned along the axis which connects the two centers. The overall elongation *L* is defined as the sum of helical rises along the ten central base pairs, corresponding to the contour length of the brown zigzag line depicted in the figure. The backbone of the duplex is represented as green ribbons.

Following standard protocols, the system was first energy-minimized in 5000 steps with restraints applied on the duplex followed by other 5000 steps of unrestrained minimization. Then, the system was heated by linearly increasing the temperature from 0 to 300 K in 300 ps at a constant volume. This was followed by an equilibration phase in NPT conditions (*T* = 300 K, *p* = 1 atm) of 20 ns. The last snapshot of the equilibration phase was employed as a starting state for the production simulations. In the case of the simulations employing the bsc1 and OL15 force fields, the duplex was stretched by a constant force in the *NVT* ensemble (*T* = 300 K) according to the protocol introduced in ref (48). This protocol was applied previously in simulations employing the bsc0 modification and focused on the same sequences as in this work,^49^ thus enabling to include in our comparison also the results stemming from the use of this older force field. The pulling force was applied between the geometric centers of C1′ atoms in the sugars within the second and second-to-last base pairs (yellow beads in Figure 1). For each sequence, five different production simulations were performed at pulling forces equal to 1, 5, 10, 15, and 20 pN. In order to compute the persistence length, we further considered production simulations in the absence of a pulling force.

In the case of the simulations employing the CHARMM36 force field, we found the sequences to be highly unstable on the microsecond time scale, in agreement with previous reports.^62,71^ To improve stability, we introduced weak interstrand bonds (4 kcal/mol Å^2^, connecting the N1 atom of a purine to the N3 atom of the paired pyrimidine) at the end base pairs to prevent fraying events, as done previously.^72^ Despite this restraint, the application of the pulling protocol led to disruption of the double helix. Therefore, for the study of this force field, we performed production simulations only in the absence of a pulling force, with the addition of further interstrand restraints when needed (see Section S6 in the Supporting Information for further details).

In the production phase, the simulation time was at least equal to 1 μs, leading to a cumulated production time of more than 170 μs. The state of the system was saved every 1000 steps. All simulations were performed with the GPU-accelerated program pmemd.cuda in the AMBER18 suite.^63^

### 2.2 Analysis

The software CPPTRAJ^73^ was used to extract the structural parameters according to the 3DNA definition.^74^ From them, we identified the overall extension *L* as the sum of the helical rises and the global torsion θ as the sum of the helical twists. In all cases, the handles were discarded from the analysis, so that only the ten central base pairs were considered (Figure 1). As shown in Figure S1 in the Supporting Information for some representative cases, the large simulation time ensured a nice convergence of these variables.

In order to characterize the elasticity of dsDNA, we describe it as an elastic rod which can be stretched and twisted. Thermally induced bending is neglected at this stage thanks to the reweighting approach described in the next section, which takes advantage of the short length of the molecules to remap the simulations onto pulling forces applied directly to the contour length of the duplex. The energy of the system thus reads
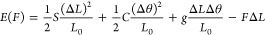
1where Δ*L* = *L* – *L*_0_ and Δ*θ* = θ – θ_0_, with *L*_0_ and θ_0_ referring to the equilibrium values of *L* and θ in the absence of mechanical stress, while *F* is the value of the pulling force. Bending fluctuations were instead analyzed in the simulations with *F* = 0, as detailed below.

#### 2.2.1 Free-Energy Perturbation

Since we are interested in the elastic properties of the central fragment, in eq 1, we conjugate the force *F* with the change of the extension Δ*L*, i.e., of the contour length of the zigzag line represented in Figure 1. However, the simulation protocol is actually applying a couple of constant forces along the line joining the centers of the second and second-to-last base pairs (yellow beads in Figure 1) so that in the simulations, the variable conjugated to *F* is their euclidean distance *d*. Hence, the energy E̅ regulating the conformational space being explored in the simulations is given by *E̅*(*F*) = *E*(*F*) – *F*(Δ*d* – Δ*L*). In order to properly sample the ensemble corresponding to eq 1, we thus employ the free-energy perturbation (FEP) technique.^75^ For any observable *O*, its average value ⟨*O*⟩_*F*_ in the ensemble corresponding to the energy *E*(*F*) can be written as
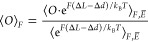
2where *k*_B_*T* is the thermal energy and  denotes averaging in the ensemble corresponding to the energy *E̅*(*F*). Note that the practical application of the FEP technique relies on the assumption of a significant overlap between the conformational spaces corresponding to *E* and *E̅*. In the present case, this is indirectly supported by the observation that the widths of the distributions of *L* and θ are significantly larger than the shift introduced by the application of different pulling forces (see Figure S1 in the Supporting Information for a representative example), thus suggesting that a similar overlap occurs in the ensembles of *E*(*F*) and *E̅*(*F*) at a given force.

In practice, eq 2 implies computing the averages from the simulations by weighting each snapshot according to the Boltzmann weight exp[*F*(Δ*L* −Δ*d*)/*k*_B_*T*]. For each observable and for the corresponding fluctuations, the error was estimated by block analysis.^75^ For each block size, the error associated with the sample was computed by bootstrapping with 1000 repetitions, where each element was extracted with a probability proportional to the corresponding weight. The weight of a block was computed by summing the FEP Boltzmann weights of the snapshots included therein. An example of the error estimation according to block size is reported in Figure S2 in the Supporting Information. The final value of the error was obtained by averaging over the last 200 block sizes.

#### 2.2.2 Effective Stretch Modulus and Crookedness Stiffness

The average change in extension ⟨Δ*L*⟩_*F*_ can be computed from eq 1, leading to^48^, where  is the effective stretch modulus. Assuming  to be independent of the pulling force, its value can thus be determined from the slope of ⟨Δ*L*⟩_*F*_ as a function of *F*.

The crookedness β is a structural parameter quantifying the displacement of the base-pair centers from the helical axis and is defined as cos β = *L*/*∑*_*i*_*u*_*i*_, where *u*_*i*_ is the center-to-center distance between base pairs *i* and *i* + 1, and the sum runs over the fragment being analyzed.^49^ The crookedness stiffness *k*_β_ describes the response of β to the pulling force and is defined in analogy to the effective stretch modulus as ⟨cos β⟩_*F*_ = *c*_0_(1 + *F*/*k*_β_), where *c*_0_ is the extrapolation of ⟨cos β⟩_*F*_ at zero force.

The values of *S̃* and *k*_β_ were computed by performing a fit of ⟨Δ*L*⟩_*F*_ and ⟨cos β⟩_*F*_ vs *F* according to the equations above. The associated errors were obtained as , where δ_ave_ is the error arising from the standard least-squares minimization applied to the average values and δ_ind_ is the error originated from the indeterminacies of the various points being fitted, estimated according to the protocol described in ref (76). This approach was also used in all the other fits performed in this work.

The values of *k*_β_ obtained for the various systems show approximately exponential behavior when plotted as a function of ⟨β⟩_0_. The function *k*_β_(⟨β⟩_0_) was thus fitted according to the formula , where *A*, *B*, and *D* are the adjustable parameters.^49^ According to a model proposed in the literature,^49^ the overall stretching response regulated by *S̃* can be ascribed to the combined effect of base-pair center alignment (decreased crookedness) and stretching of the center-to-center distance *u*_*i*_
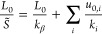
3where *k*_*i*_ and *u*_0,*i*_ are the elastic constant and zero-force extrapolation determining the response of  to the pulling force: . By following the same procedure as in ref (49), we determined the values of *k*_*i*_ and *u*_0,*i*_ for the ten different kinds of steps (Tables S1 and S2 in the Supporting Information). The predicted value of *S̃* was finally computed by means of eq 3, where *k*_β_ was obtained from ⟨β⟩_0_ via the exponential fit, and the associated indeterminacy was estimated by error propagation.

Finally, based on eq 3, the contribution to the effective stretching stemming from the crookedness can be estimated as .

#### 2.2.3 Computation of Force-Dependent Elastic Parameters

Following the theory presented in ref (51), the elastic parameters in eq 1 can be computed at each value of *F* by analyzing the fluctuations involving *L* and θ, thus unveiling the presence of force dependence in the elastic response. For completeness, we report here the formulas employed in this work and derived in ref (51)
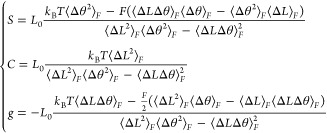
4To succinctly characterize the evolution of the elastic parameters with the external force, we perform the linear fits *S*(*F*) = *S*_0_ + *F*·d*S*/d*F*, *C*(*F*) + *C*_0_ + *F*·d*C*/d*F*, and *g*(*F*) = *g*_0_ + *F*·d*g*/d*F*, where the error on the adjustable parameters is computed as in Section 2.2.2.

#### 2.2.4 Computation of Elastic Parameters in the Absence of a Force

In the case of the simulations performed with CHARMM36 (i.e., without the presence of a pulling force), the various constants were computed by standard analysis of covariances. Particularly, the effective stretch modulus was obtained as , while the elastic constants *S*, *C*, and *g* were obtained by setting *F* = 0 in eq 4.

Finally, for all the DNA force fields considered, the persistence length *l*_p_ was computed by considering the fluctuations of the generalized tilt (τ) and roll (ρ) angles within the framework of the length-dependent elastic model.^54,60^ Particularly, we computed the persistence lengths associated with the two bending modes as  and , where *L̅* is the contour length of the line joining the base-pair centers of the central fragment, while the generalized tilt and roll were computed considering the first and last base pair within the analyzed fragment. The overall persistence length is then obtained as the harmonic mean of *l*_p_^τ^ and *l*_p_^ρ^, i.e., 1/*l*_p_ = (1/*l*_p_^τ^ + 1/*l*_p_^ρ^)/2.

## 3 Results and Discussion

We performed simulations employing the AMBER force field parm99, with either the bsc1 or the OL15 modifications. Moreover, we also considered the CHARMM36 force field. To investigate the importance of the sequence, we considered seven different molecules, as reported in the Methods. Note that this same set of sequences has been studied in the past with the bsc0 modification and with the same protocol,^48,49^ enabling throughout this work a direct comparison between the four different force fields in the same context. The sequence RNG contains all possible combinations of base-pair steps and has been studied in the past to mimic the behavior of long, random sequences employed in single-molecule experiments.^46,48^ The other six sequences are obtained as pentamers of the steps AA, AC, AG, AT, CG, or GG, which encompass the ten distinct base-pair steps.^49^ We refer to these sequences as poly-XY, where XY is any of the six steps employed to build the pentamers. The endorsement of the results obtained for these sequences as being representative of the underlying step must be however observed with a critical eye, as the flexibility of a step is strongly affected by the neighboring sequences.^77−79^

For each sequence, after standard minimization and equilibration phases, we performed pulling simulations where two opposite, constant forces are applied to the ends of the molecule (see Methods). We run simulations at different magnitudes *F* of such forces, namely 1, 5, 10, 15, and 20 pN, as well as in the absence of applied mechanical stress. Due to stability issues (see Section S6 in the Supporting Information), only the case *F* = 0 was considered in the case of CHARMM36. For the whole set, two external handles were added to the fragment of interest so as to minimize end effects. In all the subsequent analyses, only the central 10 base pairs were considered.

### 3.1 Effective Stretch Modulus Depends on the Force Field

**Figure 2:**
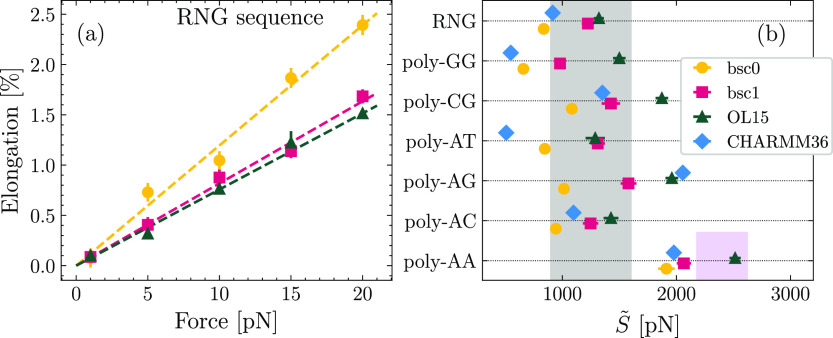
(a) Representative elongation vs force curve for the RNG sequence. The slopes of the linear fits (dashed lines) correspond to . (b) Values of  simulated for each sequence and force field. The shaded regions indicate the experimental range for random sequences^20−23^ (gray) and phased A-tracts^4^ (pink).

The first elastic parameter investigated is the effective stretch modulus *S̃*, which can be obtained from the slope of the average change in elongation ⟨Δ*L*⟩_*F*_ as a function of the applied force *F* or, in the case of CHARMM36, by analizing its fluctuations (Sections 2.2.2 and 2.2.4). As a representative case, in Figure 2a we report the stress-vs-strain curve for the sequence RNG and the three different AMBER force fields. The slope of the curve is equal to 1/*S̃*, indicating a softer response in the case of bsc0 with respect to the other modifications. In Figure 2b and in Table 1, we report the results obtained throughout the whole set. In the figure, the shaded gray region corresponds to the experimental values obtained in the literature in pulling experiments of large molecules, where a similar range of forces has been applied. To exclude the possibility of a significant dependence of our results on the TIP3P water model^66^ employed in this work, we repeated some of the simulations using the OPC model,^68^ finding small or no changes (Figure S4 in the Supporting Information). Similarly, no significant change was observed when comparing counterion neutralizing conditions (considered throughout this paper) with the case of 100 mM of added monovalent salt (Figure S5 in the Supporting Information), ensuring the minor relevance of salt screening on our results.

**Table 1:** Values of  in pN for the Various Sequences and Force Fields

force field	RNG	poly-AA	poly-AC	poly-AG	poly-AT	poly-CG	poly-GG
bsc0	837 ± 36	1912 ± 72	943 ± 47	1014 ± 39	846 ± 35	1083 ± 43	658 ± 17
bsc1	1224 ± 53	2065 ± 62	1248 ± 68	1580 ± 69	1310 ± 63	1426 ± 81	980 ± 30
OL15	1320 ± 44	2514 ± 49	1426 ± 65	1960 ± 56	1288 ± 84	1872 ± 55	1500 ± 55
CHARMM36	915 ± 20	1978 ± 34	1098 ± 31	2055 ± 17	540 ± 29	1351 ± 18	548 ± 18

From Figure 2b and Table 1, a clear pattern emerges for the AMBER family of force fields, according to which the values of *S̃* are systematically ranked in the order bsc0 < bsc1 < OL15, thus outlining a clear difference in the stiffness predicted by the various force fields. In contrast, CHARMM36 can display the stiffest (poly-AG) or the softest response (poly-AT and poly-GG) according to sequence, as well as intermediate values of the elastic constant (RNG, poly-AA, poly-AC, and poly-CG).

At a general level, the values obtained for bsc0 and bsc1 mostly lie within the experimentally known range, while for OL15 they are usually found to be larger. Similarly, for CHARMM36, some outliers can be observed. While this suggests that OL15 and CHARMM36 might be less precise in capturing dsDNA elongation elasticity, one should take this indication with some caution, as the sequences poly-XY are not directly comparable with long, random sequences usually employed in single-molecule experiments. In this regard, the most valuable system is provided by the random-like sequence RNG, for which the four force fields predict values of *S̃* within the experimental range. Interestingly, in all cases the sequence poly-AA is found to be significantly stiffer than the other molecules (with the exception of poly-AG in the CHARMM36 force field). The most direct experimental comparison for this system is provided by the phased A-tracts reported in a recent study,^4^ where pulling by optical tweezers estimated *S̃* = (2400 ± 220)pN. This is in line with the three predictions, with the best quantitative agreement being found for OL15.

### 3.2 Stretching is Determined by Crookedness

The crookedness β is a structural parameter characterizing the displacement of the base-pair centers from the helical axis.^49^ By definition, larger values of β indicate a more crooked structure, with β = 0 corresponding to perfectly aligned centers (see Methods for the formal definition). It was proposed that the elongation response of dsDNA to a pulling force can be described as the combined effect of force-induced alignment of the centers and stretching of the center-to-center distance between consecutive base pairs.^49^ Intriguingly, this led to a model which, in the case of bsc0, quantitatively predicted the value of *S̃* from knowledge of β in the absence of mechanical stress, combined with tabulated values of stiffness for the ten possible base-pair step combinations. In other terms, this allows predicting the mechanical response from the sequence of the dsDNA fragment and from knowledge of its structure in the absence of applied force.

**Figure 3:**
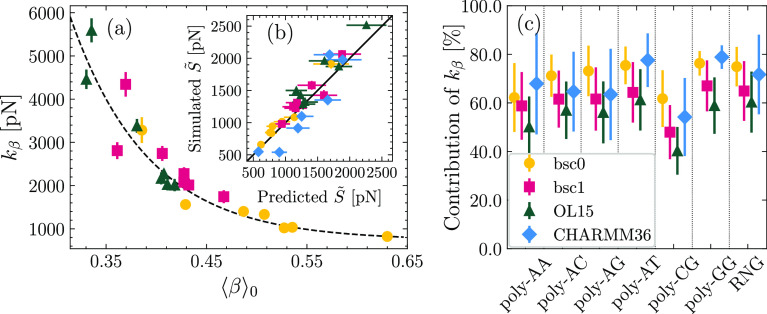
(a) Elastic constant associated with crookedness, *k*_β_, as a function of the average crookedness in the absence of applied force, ⟨β⟩_0_. Dashed line is a fit to the function . (b) Comparison between predicted values of *S̃* and results obtained from simulations. (c) Contribution of *k*_β_ to the overall stretching response.

We employed the extended data set to check whether this model is also applicable to the other force fields, or rather, whether it is restricted to bsc0. Following ref (49), we first characterized the stiffness *k*_β_ associated with β, which accounts for the energetic cost needed to align the centers (see Methods for the formal definition). In Figure 3a, we report the values of *k*_β_ as a function of the average crookedness ⟨β⟩_0_ obtained in the unperturbed case. Remarkably, the data set corresponding to the three AMBER force fields (bsc0, bsc1, and OL15) can be nicely fitted by a single function of the form . This phenomenological functional form was proposed in ref (49) for the bsc0 data and is found here to be able to capture simultaneously the data for the three force fields. The values of the optimized parameters are *A* = (0.29 ± 0.07)·10^6^ pN, *D* = 12.8 ± 0.7, and *B* = (736 ± 37)pN. This emerging pattern suggests that the difference in rigidity of the various AMBER modifications can be ultimately ascribed to the improved structural features introduced in bsc1 and OL15, at least at the level of the crookedness stiffness. The crookedness constants corresponding to CHARMM36 were not computed since only simulations of unperturbed molecules were performed in this case.

As the next step, we employed the simulation results for poly-XY to determine the elastic constants associated with the ten distinct base-pair step combinations^49^ (reported in Table S1 in the Supporting Information). In contrast with the case of *k*_β_, inspection of the step stiffness constants did not reveal a clear pattern in their dependence on the chosen AMBER force field. This further supports the idea that it is the change in structure (i.e., the unperturbed crookedness) which ultimately originates the ranking bsc0 < bsc1 < OL15 observed for *S̃* in Figure 2b and Table 1.

Building on this point, we consider the model from ref (49), which predicts the value of *S̃* by considering an effective series of springs involving the crookedness rigidity and the center-to-center distances (see Methods). The value of *k*_β_ is computed from the crookedness of the unperturbed system via the empirical exponential function. As for the steps, given the absence of a clear force-field dependence, and with the aim of building a theoretical framework including the three modifications at the same time, we consider for the elastic parameters the averages of the values obtained for bsc0, bsc1, and OL15 (see Section S2 in the Supporting Information). In Figure 3b, we compare *S̃* as predicted by the model and the values retrieved directly from the simulations. The close agreement between prediction and numerical results further endorses the universality of the model across the spectrum of the different AMBER force fields.

The comparison between model prediction and simulation results for *S̃* can also be used as an indirect proxy to check whether this universality also applies to CHARMM36. In this regard, in Figure 3b we include data points corresponding to CHARMM36, where the predicted value of *S̃* was computed assuming that the formula  holds also for CHARMM36 with the same parameters determined for the AMBER force fields. Although the points are somewhat noisier than the rest of the data, we obtain a remarkable quantitative agreement between predicted and computed values. We stress that these predictions for the CHARMM36 elasticity are based on parameters obtained solely from the analysis of the AMBER simulations. This quantitative agreement thus strongly supports the existence of a single law dictating the stretching elasticity of dsDNA starting from its crookedness, irrespective of the force field being employed.

Based on the model, we further investigated the contribution of *k*_β_ to *S̃*,^49^ which was estimated as  (see Methods) and is reported in Figure 3c. In most cases, crookedness is the major factor responsible for the response to the pulling force,^49^ although from a quantitative perspective, its contribution is lower for bsc1 and OL15. This is in line with the absence of a pattern in the step stiffness constants, which, once combined with the stiffening of *k*_β_ induced by the enhanced spontaneous alignment of the centers, results in an overall smaller contribution of the crookedness stiffness.

### 3.3 Stretch Modulus Increases with Force

The effective stretch modulus *S̃* quantifies the net elongation response to the pulling force. Due to a coupling between the extension *L* and the torsion θ of the duplex, the net elongation change ⟨Δ*L*⟩_*F*_ induced by *F* results from the interplay between these two degrees of freedom. Quantitatively, one finds that , where *S* is the stretch modulus, *C* is the twist modulus, and *g* is the twist-stretch coupling (see Methods for further details).

**Figure 4:**
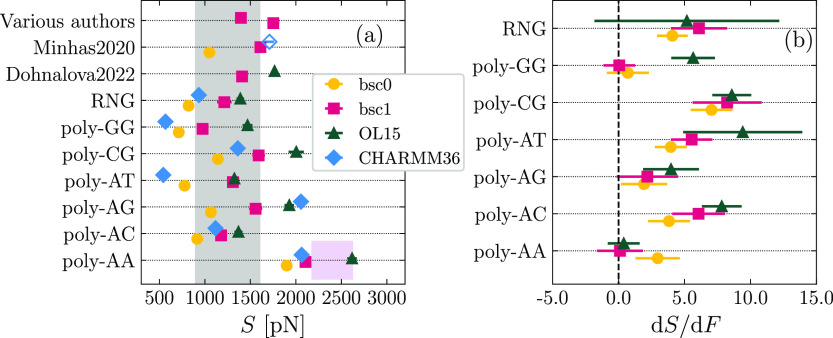
(a) Values of *S* in the absence of pulling force computed by analysis of fluctuations. The empty diamond for the data set “Minhas2020” corresponds to simulations performed in the literature with CHARMM27.^62^ The shaded region corresponds to the experimental range. Due to the lack of direct experimental determination of *S*, the shaded region corresponds to the values of *S̃* measured in the literature, as in Figure 2b. (b) Slope of the *S*(*F*) curve obtained for the AMBER force fields.

In Figure 4a, we report the values of *S* obtained by fluctuations in the absence of a pulling force. The data show a strong resemblance to the values of *S̃* reported in Figure 2b. This is expected since the typical values of *g* and *C* (see below) provide a small correction when employing the formula , thus leaving unchanged the pattern observed above, i.e., the systematic ranking bsc0 < bsc1 < OL15. For completeness, we also report the values of *S* published in the literature.^50,57,61,62^ Particularly, the data labeled “Dohnalova2022” consider the same sequence for bsc1 and OL15,^50^ confirming for a specific case our general observation on the higher rigidity of OL15. Similarly, the data set “Minhas2020” is also in line with our results, showing that for the same sequence, bsc1 is more rigid than bsc0.^62^ In this case, the CHARMM27 force field was also studied, yielding a value of *S* (empty diamond in Figure 4a) similar to the one predicted by bsc1. On a more general level, we note that the various results from the literature further support the robustness of our results with respect to the choice of water and ions details, as some of the reported works have employed different conditions as the ones considered here, such as the SPC/E model for water,^50,57^ potassium ions modeled via the Dang model^50^ (at difference with the Joung–Cheatham parameters for sodium employed here),^50^ as well as different concentrations of salt ranging from neutralizing conditions^57^ up to 150 mM.^50^

The description of dsDNA via only two variables is necessarily an effective one, so that the elastic parameters *S*, *C*, and *g* are expected to change according to the applied mechanical stress.^51^ We have recently introduced a theoretical framework which allows us to determine the force-dependent values of the elastic parameters by studying their fluctuations in the constant-force ensemble and employing it to analyze the force-dependent behavior of dsDNA and dsRNA.^51^ For dsDNA, in our previous work, we employed the bsc0 data. Here, we study the extended data set to check the robustness of our conclusions with respect to the force field considered. For completeness, we report in the Methods the formulas derived in ref (51) which are employed in the present study (eq 4). Since for CHARMM36 we performed simulations only for *F* = 0 pN, this force field was not included in this analysis.

To characterize the force-induced change in the stretch modulus, we performed a linear fit of *S*(*F*), for which the slope d*S*/d*F* is reported in Figure 4b. For all sequences, d*S*/d*F* ≥ 0, indicating that dsDNA stiffens upon pulling, in agreement with our previous report on bsc0.^51^ Microscopically, this stiffening can be ascribed to the progressive alignment of the aromatic rings of consecutive base pairs due to the action of the force, which increases the strength of stacking interactions.^51^ This picture is supported quantitatively by the significant correlation (*r* ≃ 0.70) between the change in stretch modulus d*S*/d*F* and the change in slide dλ/d*F*, as can be observed from Figure S3 in the Supporting Information. All in all, the present analysis fully extends to the bsc1 and OL15 force fields our previous findings on the force-induced stiffening of dsDNA.^51^

### 3.4 Twist Modulus Ranking Depends on Sequence

**Figure 5:**
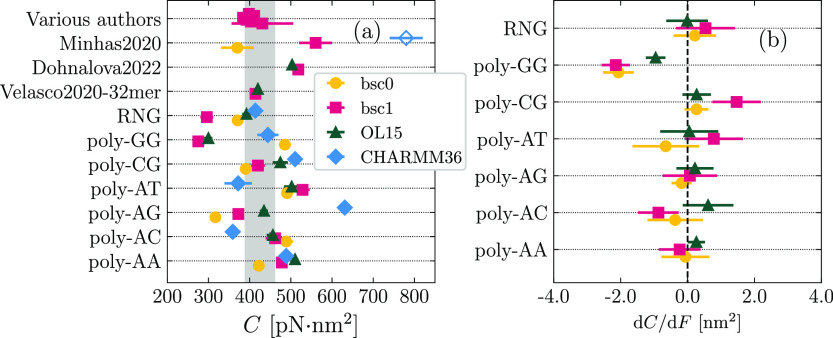
(a) Values of *C* in the absence of pulling force computed by analyzing fluctuations. The empty diamond for the data set “Minhas2020” corresponds to simulations performed in the literature with CHARMM27.^62^ The shaded region corresponds to the experimental range. (b) Slope of the *C*(*F*) curve obtained for the AMBER force fields.

Our results for the twist modulus *C* are reported in Figure 5. In Figure 5a, we show the values of *C* obtained in the absence of a pulling force, together with data collected from the literature.^50,57,60−62^ The agreement with the experimental values (shaded area) is generally good for all the force fields. When considering random-like sequences (first five rows in Figure 5a), bsc1 yields results quantitatively within the experimental range in several cases (“Various authors”^57,60,61^ and “Velasco2020-32mer”^60^), although other works report larger values of *C* (“Minhas2020”^62^ and “Dohnalova2022”^50^), while the random sequence RNG studied in this work displayed a softer twist response. The ranking bsc0 < bsc1 < OL15 holds for several sequences (poly-AA, poly-AG, poly-CG, and RNG) but is not as general as we found for *S*. For instance, for poly-AT OL15 is found to be as soft as bsc0, while bsc0 shows the highest twist stiffness in the case of poly-AC. The most peculiar case is provided by poly-GG, for which bsc0 has a much larger value of *C* than both the other modifications, despite showing enhanced flexibility in the stretching mode (Figure 4a). This case reflects previous observations on the dependence of rigidity on the elastic mode under consideration.^80,81^ The lack of a general pattern in *C* is supported by other data from the literature, which show reversed rankings for bsc1 and OL15 (OL15 < bsc1 in ref.,^50^ bsc1 ≲ OL15 for the 32mer in ref (60)). As for CHARMM36, similarly to the case of *S* and *C*, there is no particular tendency when compared to the AMBER force fields; for instance, CHARMM36 has a markedly stiffer twist response for poly-AG but enhanced flexibility in the case of poly-AC. Notably, a previous report employing CHARMM27 (empty diamond in “Minhas2020”^62^) has found a 2-fold increase of *C* as compared to the experimental range.

In Figure 5b, we report the slope d*C*/d*F* to characterize the force dependence of *C*. We observe virtually no change in the twist modulus, with the exception of the sequence poly-GG, which shows a clear negative slope for all cases. It is worth mentioning that this duplex is the most crooked molecule, for which the force-induced straightening of the spontaneous curvature is likely to induce a softer twist response.^51^

### 3.5 Force Fields Qualitatively Capture the Twist-Stretch Coupling

**Figure 6:**
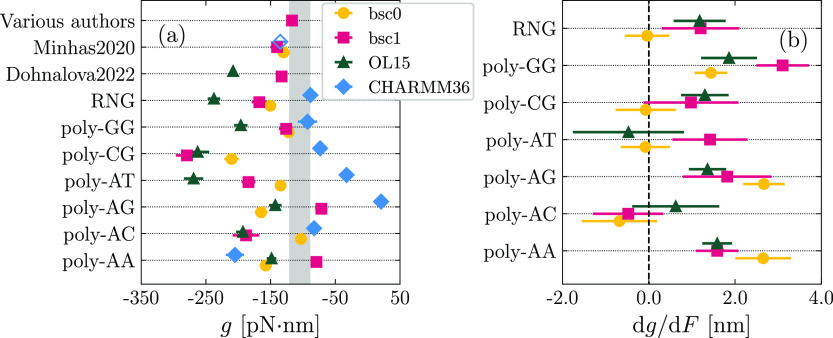
(a) Values of *g* in the absence of pulling force computed by analyzing fluctuations. The empty diamond for the data set “Minhas2020” corresponds to simulations performed in the literature with CHARMM27.^62^ The shaded region corresponds to the experimental range. (b) Slope of the *g*(*F*) curve obtained for various sequences and force fields.

In Figure 6, we report the results obtained for the twist-stretch coupling *g*. In almost all cases, we find that *g* < 0 (Figure 6a), in agreement with experimental results.^26^ Quantitatively, there is a tendency for all the AMBER force fields to overestimate the magnitude of *g* with respect to experiments (shaded region in Figure 6a), as observed in previous work,^48^ while CHARMM36 tends to underestimate the coupling. While this feature might be a potential target to address in future refinements of the force fields, such mismatches must be taken with caution due to the likely dependence of *g* on the length of the fragment under consideration.^76^ Particularly, based on a nucleotide-level coarse-grained model, we have previously reported significant changes of *g* in the 20–40 base-pairs range,^76^ so that such an effect is expected to be further enhanced for the large molecules employed in experiments. In terms of ranking, in analogy to *C*, no clear patterns are present, although one may notice a generic shift of OL15 values toward larger magnitudes of *g*.

In Figure 6b, we report the slope d*g*/d*F* characterizing the change of twist-stretch coupling with force. For all force fields and all sequences, one has d*g*/d*F* ≥ 0 within error, indicating a weakening of *g* with the pulling force. Again, this is in line with previous reports on bsc0^48^ and with the experimental literature.^26,28^ As a future direction, it will be an interesting challenge to check the performance of the various force fields in capturing the experimentally observed sign reversal of *g* at forces around 40 pN.

### 3.6 Persistence Length is Overestimated by All Force Fields

**Figure 7:**
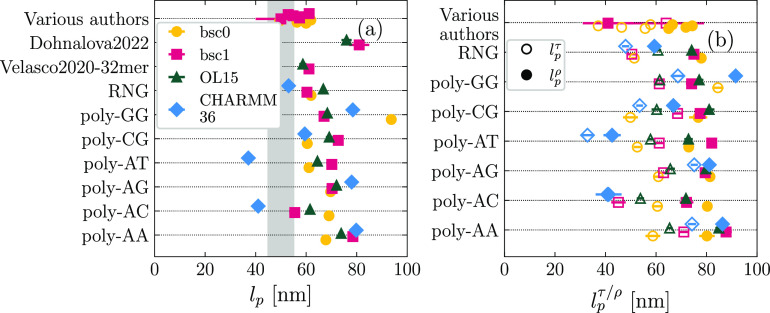
(a) Values of overall persistence length *l*_p_ computed for the central decamer of each sequence. The shaded region corresponds to the experimental range. (b) Contribution to the persistence length coming from generalized roll (*l*_p_^ρ^, full symbols) and tilt (*l*_p_^τ^, empty symbols), computed on the central decamer.

In Figure 7, we report the results obtained for the persistence length, computed by analyzing the fluctuations of the generalized roll and tilt angles as defined by the length-dependent elastic model,^54,60^ by considering the global bending of the central decamer of each sequence (see Methods). At a general level, all the force fields tend to overestimate the overall persistence length *l*_p_ (Figure 7a). Remarkably, for the random sequence RNG, CHARMM36 shows the best agreement with experiments (53 ± 1 nm, within the experimental range). To our knowledge, there have been no previous attempts in the literature to compute *l*_p_ from atomistic simulations with CHARMM36; hence, whether this observation stands for a general behavior on random sequences will need to be confirmed in future works extending the pool of simulated molecules. We observe no general tendency for the AMBER force fields, for which all possible rankings are present.

It is worth discussing shortly the various outliers. CHARMM36 predicts soft bending for poly-AC and poly-AT, which we also found to be the most unstable sequences in this force field (see Section S6 in the Supporting Information). Although we filtered out transient disruptions of the duplex, the weakness of the stacking interactions likely allows conformational changes, promoting the thermally induced bending of the molecule. A second remarkable outlier is poly-GG within the bsc0 modification, for which the persistence length is roughly twice (94 ± 1 nm) the value obtained experimentally for random sequences. This high bending stiffness, together with the large crookedness (Figure 3a) and the small stretching modulus (Figure 4a), is reminiscent of the typical elastic behavior of double-stranded RNA,^23^ appointing the sequence poly-GG—as described by bsc0—as an intermediate structure between ideal B-DNA and A-DNA, as suggested previously based on crookedness data.^49^ Nevertheless, the twist-stretch coupling of poly-GG is still negative, thus retaining the standard behavior of dsDNA in terms of the positive correlation between extension and torsion, which has an opposite sign for double-stranded RNA.^23^

In Figure 7b, we focus on the anisotropy of the bending stiffness, considering the persistence length *l*_p_^ρ^, associated with the roll angle (i.e., bending toward the grooves, full symbols in Figure 7b), and the tilt persistence length *l*_p_^τ^, which accounts for bending toward the backbone (empty symbols in Figure 7b). Note that the overall persistence length *l*_p_ is found as the harmonic mean of *l*_p_^ρ^ and *l*_p_^τ^. No patterns are observed when comparing the force fields with respect to each other. Instead, for each sequence and force field, we systematically find that *l*_p_^ρ^ is larger than *l*_p_^τ^, in agreement with previous reports in the literature for fragments of similar length.^81^ We note that this trend is inverted if one considers local rather than global bending flexibility, for instance at the level of a base-pair step (Figure S8 in the Supporting Information), in line with the larger variance of dinucleotide roll vs tilt degrees of freedom observed both in crystal structures^82^ and simulations.^61^

## 4 Conclusions

In this work, we have performed a systematic comparison of the molecular mechanics of dsDNA as predicted by the most advanced force fields for dsDNA in the AMBER and CHARMM families, including the older gold standard provided by the bsc0 modification of the parm99 force field. We have found that the global response of the duplex to a pulling force is captured with good precision by all the models, although some clear differences are evident.

The three AMBER modifications show similar behavior in the twist modulus and in the twist-stretch coupling, in terms of both their average values and their evolution with the magnitude of the external force being applied. As for the stretching, a clear pattern is observed, according to which the force fields are ranked from the softest to the stiffest as bsc0 < bsc1 < OL15. This feature is perhaps the most prominent difference observed to date between the bsc1 and OL15 modifications, for which previous studies have reported very similar performance in reproducing the structural features of dsDNA.^58^ CHARMM36 predicts similar values for stretch and twist moduli when compared to the AMBER force fields, although the difference in flexibility is strongly dependent on the particular sequence being inspected. In contrast, for the twist-stretch coupling, it predicts values systematically lower in magnitude than any of the three AMBER variants. In terms of persistence length, all force fields tend to overestimate their value in comparison to experiments, although CHARMM36 performs particularly well in the case of a random sequence.

An intriguing conclusion of our analysis is the dominant role played by crookedness in determining the stretch behavior of dsDNA. Previous observations disclosed a mapping between the stretch modulus and the sequence-dependent conformation via the crookedness parameter, analyzing several sequences within the bsc0 force field.^49^ Our results not only extend this concept to the other AMBER force fields and to CHARMM36 but also show that a single mapping captures simultaneously the stretch elasticity of all force fields. Particularly, this allowed us to accurately predict the sequence-dependent values of the stretching modulus in CHARMM36 by means of a mapping parametrized by using as input only the AMBER simulations, thus unveiling a common mechanism originating from the different stretching responses of the four force fields.

Overall, our results highlight the presence of more marked differences between the various force fields when looking at elasticity as compared to previous structural reports, whose nature varies with the specific sequence of the fragment under inspection. Yet, all the analyzed variants perform quite well in capturing the elasticity of dsDNA. CHARMM36 appears to be less stable than the AMBER force fields, which imposes extra cautions to be taken when analyzing DNA flexibility with simulations based on this force field. As for the AMBER force fields, the differences observed for the stretching modulus suggest that bsc1 might be preferred over OL15 for studies focused on the mechanics of dsDNA. However, due to the peculiar features of the poly-XY sequences from which this difference is argued, this observation has to be taken with caution, particularly in light of the well-known dependence of dinucleotide flexibility on the sequence of the neighboring fragments.^77−79^ In order to conclusively determine the force field with the best performance, it would be ideal to couple the present results with single-molecule experiments on large poly-XY sequences, so as to provide the groundwork for a thorough and decisive comparison.

## Supporting Information Available

Convergence of simulations, step-dependent stretching stiffness, relation between changes in stretch modulus and slide, dependence of stretch modulus on water model and ionic strength, analysis of stability of simulations with CHARMM36, and anisotropy of persistence length according to global and local definitions (PDF)
