# Supplementary material for: Systematic Comparison of Atomistic Force Fields for the Mechanical Properties of Double-Stranded DNA
Source: J Chem Theory Comput. 2024 Feb 27;20(5):2261–72. doi: 10.1021/acs.jctc.3c01089 (PMC10938644; doi:10.1021/acs.jctc.3c01089)
Supplement: Supplementary file 1 — ct3c01089_si_001.pdf [file ct3c01089_si_001.pdf]

Supporting Information: Systematic comparison of atomistic force fields for the mechanical properties of double-stranded DNA

Carlos Roldán-Piñero,[†] Juan Luengo-Márquez,[†,‡] Salvatore Assenza,[*,†,‡,¶] and Rubén Pérez[*,†,¶]

†*Departamento de Física Teórica de la Materia Condensada, Universidad Autónoma de Madrid, E-28049 Madrid, Spain*

‡*Instituto Nicolás Cabrera, Universidad Autónoma de Madrid, E-28049 Madrid, Spain*

¶*Condensed Matter Physics Center (IFIMAC), Universidad Autónoma de Madrid, E-28049 Madrid, Spain*

E-mail: salvatore.assenza@uam.es; ruben.perez@uam.es

1 Convergence of simulations

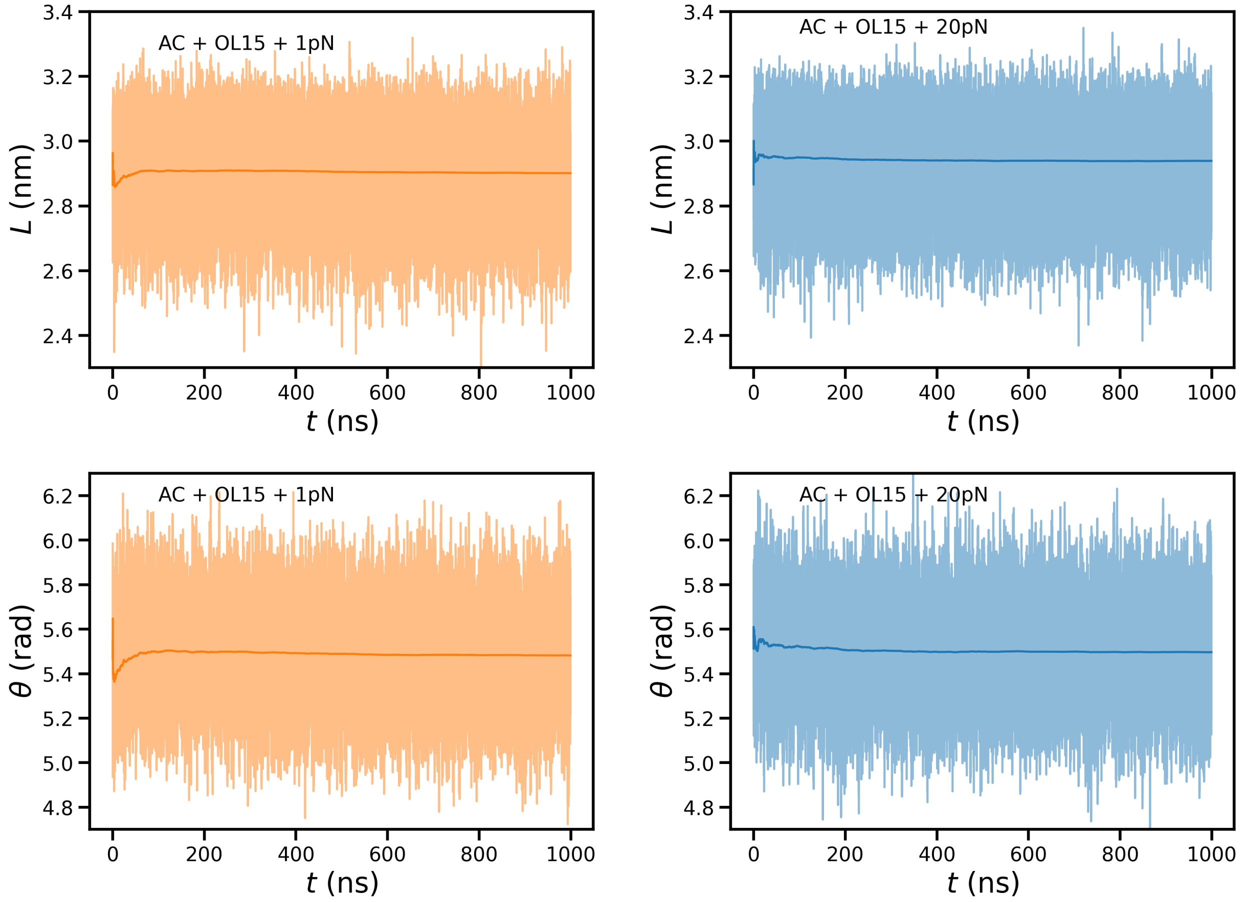

Figure S1: Representative examples of convergence for the extension L (top) and the torsion θ (bottom). Transparent lines show the trajectories $L(t)$ and $\theta(t)$, with t being the simulation time. Full lines report the running average up to time t along the simulation time. Left and right column correspond to pulling forces equal to $F = 1$ pN and $F = 20$ pN, respectively. From their comparison, one can appreciate the different magnitude between the force-induced shift of the average value and the thermally-induced fluctuations at each force.

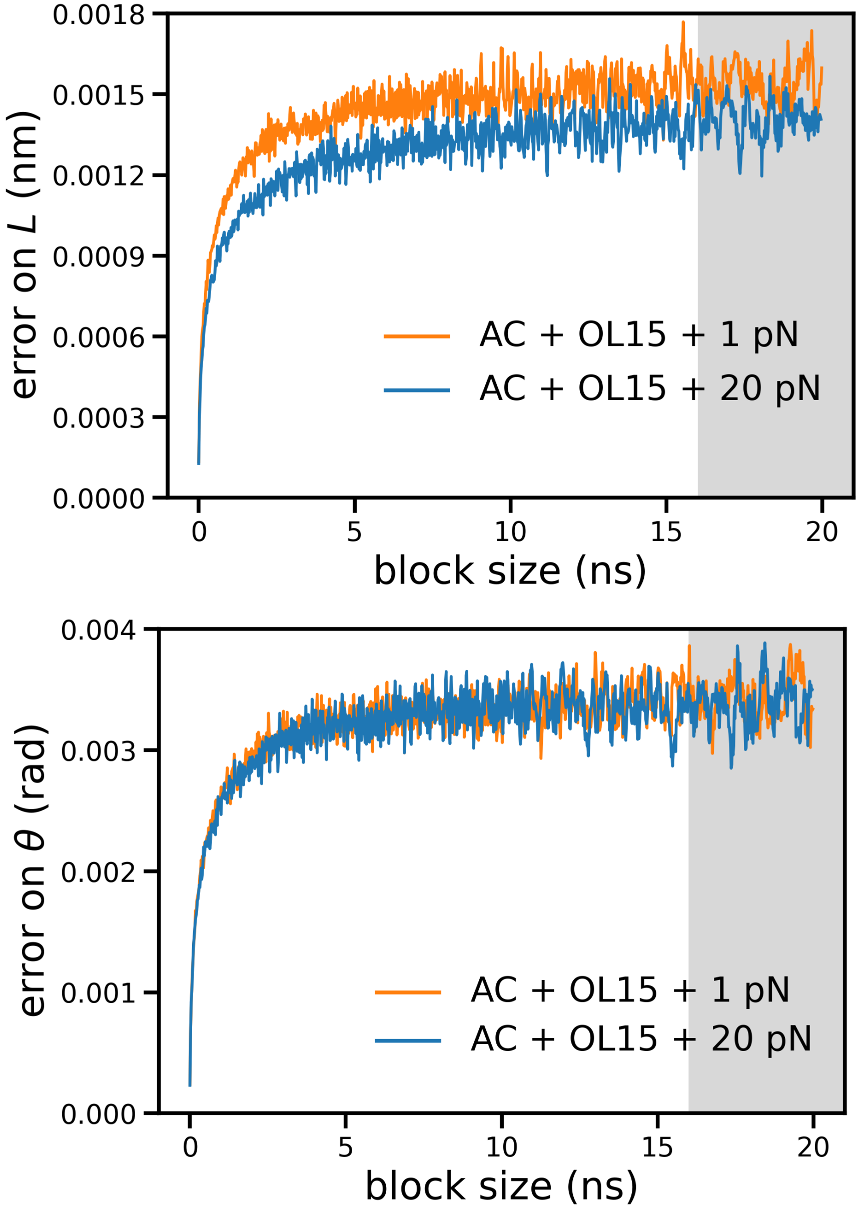

Figure S2: Representative examples of block analysis for error estimation on L(top) and θ (bottom). The final error was obtained by averaging the values of the last 200 block sizes, highlighted by the shaded region.

2 Step-dependent stretching stiffness

Table S1: Elastic constant k and unperturbed length u_0 for the 10 different kinds of steps and the AMBER force fields. For each case, the values were determined by collecting the force-dependent average extension $\langle u \rangle_F$ of all the steps of the kind under inspection from the sequences poly-XY. Then, the parameters were obtained by fitting the results via the formula $\langle u \rangle_F = u_0(1 + F/k_i)$. In the case of u_0, errors are not listed as they are smaller than the last reported figure. For some steps, the constant k is characterized by large (possibly negative) values and high error, highlighting in practice no force dependence for u (e.g. see step GA). In all cases, we observed that the change induced by the force on the average values is much smaller than the typical fluctuations of the values of u at any given force.

Force-field dependence

	bsc0		bsc1		OL15	
Step	k (pN)	u_0 (nm)	k (pN)	u_0 (nm)	k (pN)	u_0 (nm)
AA	4865 ± 365	0.352	4066 ± 393	0.352	4766 ± 227	0.346
AC	1856 ± 333	0.353	3561 ± 450	0.351	4450 ± 567	0.341
AG	2226 ± 189	0.367	2419 ± 307	0.357	3401 ± 350	0.356
AT	3187 ± 261	0.347	4061 ± 581	0.340	5599 ± 1254	0.336
CA	8137 ± 3657	0.355	2434 ± 336	0.365	4595 ± 697	0.364
CG	2721 ± 408	0.339	1903 ± 144	0.346	2678 ± 132	0.355
GA	-31176 ± 28986	0.354	19164 ± 15880	0.358	13811 ± 4640	0.347
GC	7610 ± 1532	0.359	25961 ± 23327	0.356	4785 ± 572	0.343
GG	3355 ± 207	0.379	2319 ± 226	0.369	5811 ± 821	0.359
TA	10875 ± 3628	0.363	3512 ± 575	0.374	2702 ± 442	0.374

Table S2: Elastic constant k and unperturbed length u_0 for the 10 different kinds of steps averaged over the three AMBER force fields. For each case, the values were determined from Table S1 by considering the weighted average $w_{k,\mathrm{bsc0}}k_{\mathrm{bsc0}} + w_{k,\mathrm{bsc1}}k_{\mathrm{bsc1}} + w_{k,\mathrm{OL15}}k_{\mathrm{OL15}}$ and $w_{u,\mathrm{bsc0}}u_{0,\mathrm{bsc0}} + w_{u,\mathrm{bsc1}}u_{0,\mathrm{bsc1}} + w_{u,\mathrm{OL15}}u_{0,\mathrm{OL15}}$. In the previous formula, each weight is proportional to the inverse of the squared error from Table S1, and the normalizations $w_{k,\mathrm{bsc0}} + w_{k,\mathrm{bsc1}} + w_{k,\mathrm{OL15}} = 1$ and $w_{u,\mathrm{bsc0}} + w_{u,\mathrm{bsc1}} + w_{u,\mathrm{OL15}} = 1$ hold. Errors were propagated from Table S1 as $\sqrt{\delta_1^2 + \delta_2^2}$ where, in the case of k, $\delta_1 = \sqrt{(w_{k,\mathrm{bsc0}}\delta k_{\mathrm{bsc0}})^2 + (w_{k,\mathrm{bsc1}}\delta k_{\mathrm{bsc1}})^2 + (w_{k,\mathrm{OL15}}\delta k_{\mathrm{OL15}})^2}$ and δ_2 is the weighted standard deviation of the values of k. A similar formula applies for u_0.

Average values		
Step	k (pN)	u_0 (nm)
AA	4653 ± 337	0.348 ± 0.003
AC	2821 ± 1092	0.346 ± 0.005
AG	2475 ± 457	0.360 ± 0.005
AT	3413 ± 575	0.343 ± 0.004
CA	2877 ± 995	0.362 ± 0.004
CG	2345 ± 398	0.352 ± 0.005
GA	13185 ± 8239	0.351 ± 0.004
GC	5142 ± 1172	0.351 ± 0.007
GG	2982 ± 746	0.372 ± 0.009
TA	3076 ± 917	0.367 ± 0.005

3 Relation between changes in stretch modulus and slide

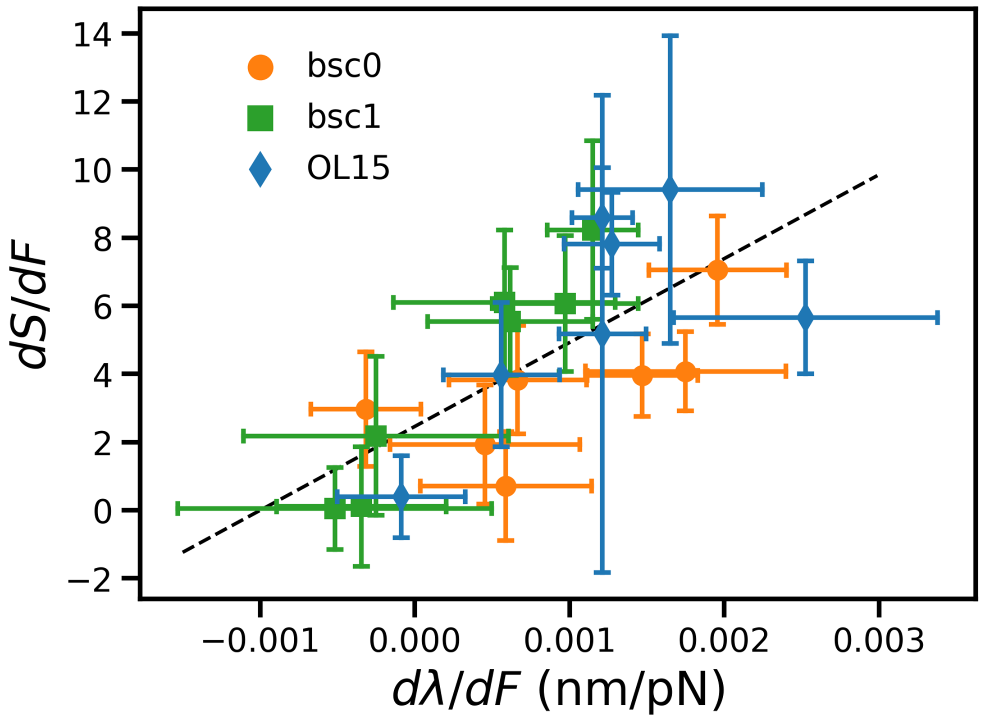

Figure S3: Variation dS/dF of stretch modulus and of slide $d\lambda/dF$ upon changing the magnitude of the pulling force F. The dashed line represents a linear fit of the whole dataset.

4 Dependence of stretch modulus on water model

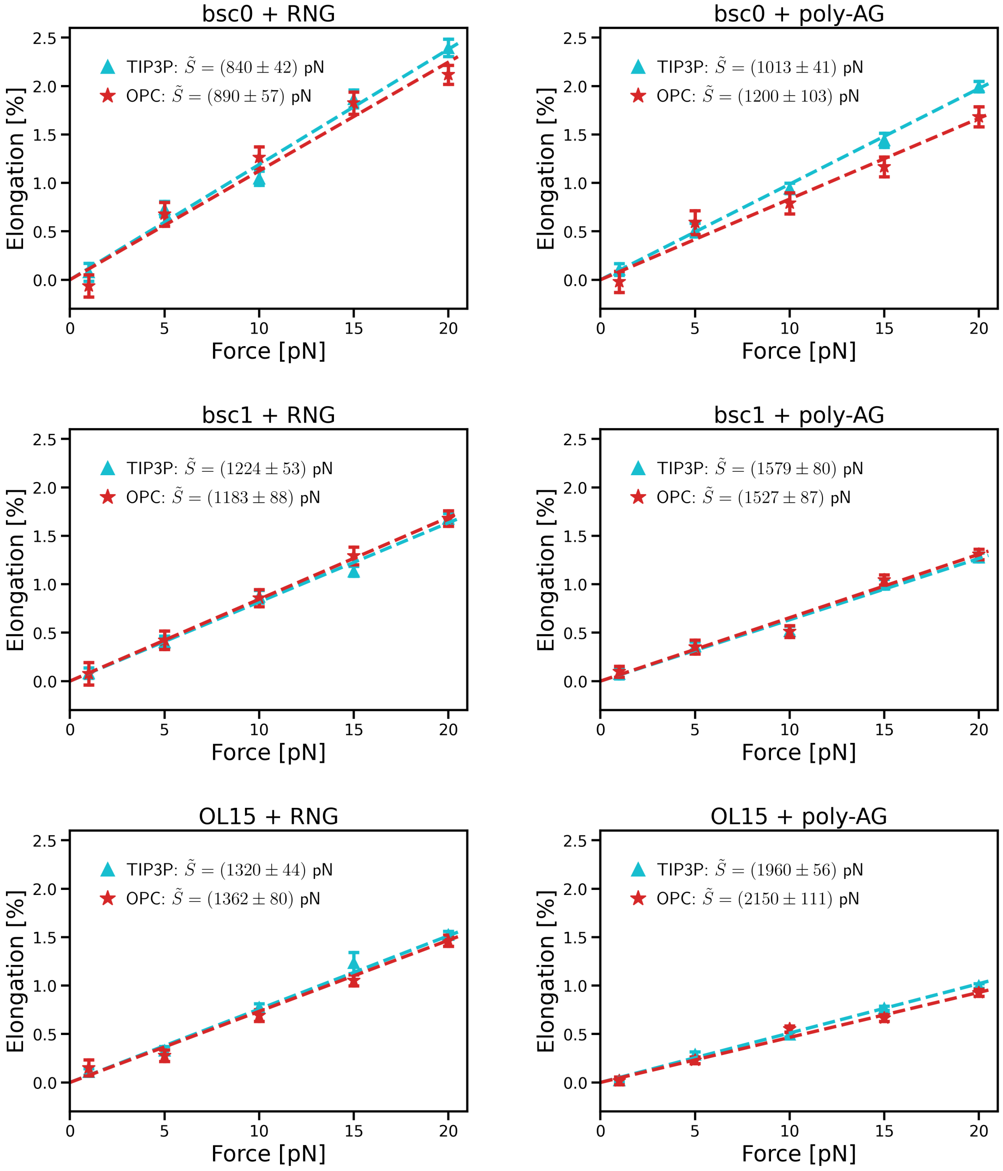

Figure S4: Elongation vs force for simulations performed with two different water models: TIP3P (cyan triangles) and OPC (red stars). We considered some representative cases corresponding to sequences RNG (left) and poly-AG (right), for various choices of DNA force fields: bsc0 (top), bsc1 (center) and OL15 (bottom). Dashed lines correspond to the linear fits from which the effective strech modulus $\tilde{S}$ was computed. The corresponding values of $\tilde{S}$ are reported in the plots. In most cases, no difference is detected, although for the combination bsc0 + poly-AG the water model OPC predicts a stiffer response. The magnitude of the change in $\tilde{S}$ (~ 18 %) is however small in comparison with the 5-fold range of sequence-dependent variability reported in Fig.2b in the main text.

5 Dependence of stretch modulus on ionic strength

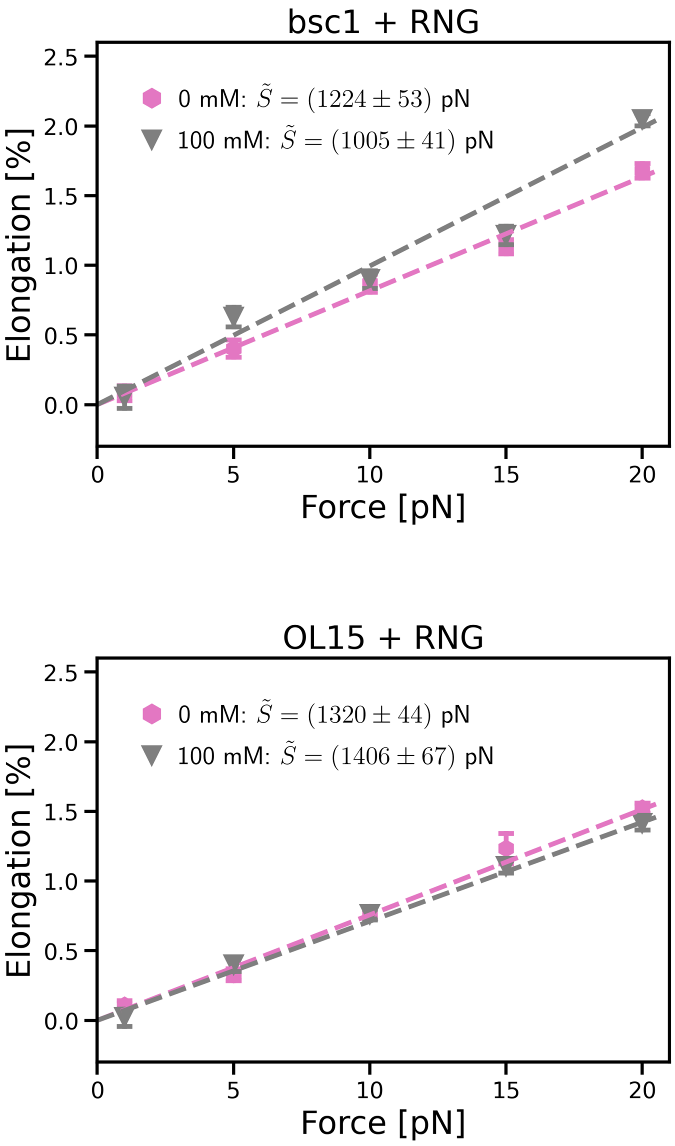

Figure S5: Elongation vs force for simulations performed with neutralizing conditions (pink hexagons) or by adding 100 mM of monovalent salt (gray inverted triangles). We considered two representative cases corresponding to sequence RNG for bsc1 (top) and OL15 (bottom). Dashed lines correspond to the linear fits from which the effective strech modulus $\tilde{S}$ was computed. The corresponding values of $\tilde{S}$ are reported in the plots. For bsc1, the salt screening induced a softer elastic response ($\sim$ 18 %), while virtually no change is detected for OL15. In any case, the magnitude of the change is small in comparison with the 5-fold range of sequence-dependent variability reported in Fig.2b in the main text.

6 Analysis of stability of simulations with CHARMM36

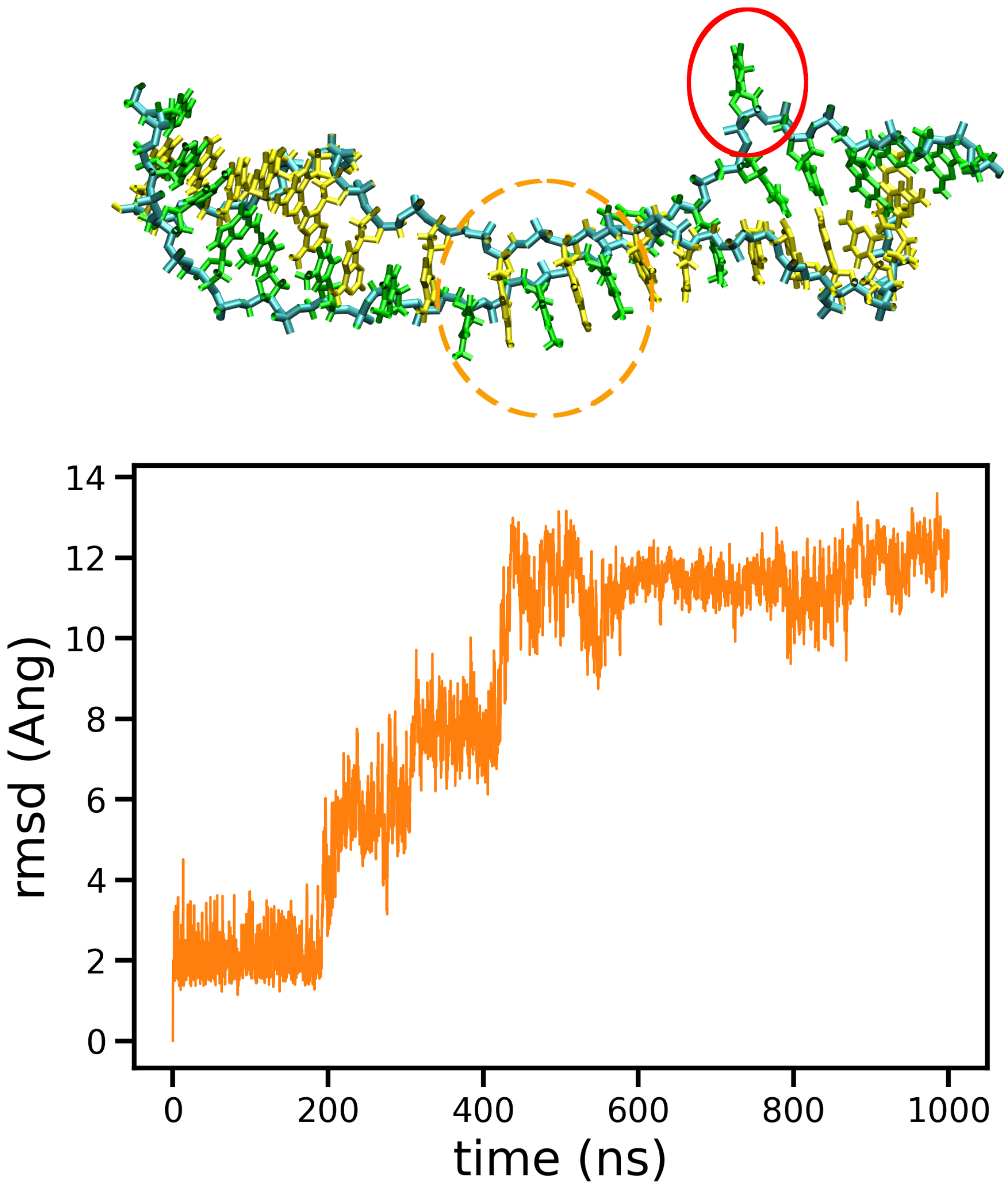

Figure S6: Top: representative snapshot showing the instability of simulations with CHARMM36 in the presence of a pulling force, corresponding to poly-AA with $F = 5$ pN. In this instance, we observe the presence of unpaired bases protruding towards the outside of the duplex (red circle) or stacked in an alternating fashion (orange circle). Bottom: time evolution of the rmsd for the same simulation as in the top panel, showing the disruption of the duplex after about 200 ns.

As mentioned in the main text, to enhance the stability of the duplex while employing CHARMM36, we introduced bonds joining paired purines (N1 atom) and pyrimidines (N3

atom). Following previous literature[S1], these restraining bonds were introduced at the end base pairs. Nevertheless, this strategy proved insufficient to ensure stability against the presence of even moderate pulling forces. As an example, in Fig. S6 we depict a snapshot obtained for sequence poly-AA under the presence of a force $F = 5$ pN (top) as well as the evolution of the rmsd (bottom).

We thus limited our simulations to the unperturbed case, where no pulling force is applied. This allowed to simulate stable duplexes for the sequences poly-AA, poly-AG, poly-CG and poly-GG, while the three remaining sequences still showed marked instability. For these sequences (RNG, poly-AC, poly-AT), we thus opted for a stronger restraint, where the stabilizing bonds were introduced for all base pairs. We ensured that this approach does not significantly affect the computed elastic constants by repeating the simulation with the full restraint also for the sequence poly-AG, obtaining – with respect to the case where only the end pairs were restrained – the same values of $\tilde{S}$ (2085 ± 17 pN vs 2055 ± 17 pN) and C (626 ± 9 pN vs 631 ± 10), and a modest change in g (-6 ± 7 pN·nm vs 21 ± 8 pN·nm). Similarly, virtually no change was detected for the persistence length (75 ± 1 vs 75 ± 1 for l_p^τ, 85 ± 1 vs 82 ± 2 for l_p^ρ, 80 ± 1 vs 78 ± 1 for l_p). With this strategy, overall stable trajectories were found, although some transient departures from the B-DNA conformation were still observable (Fig. S7). These transient conformations were discarded in the computation of the various elastic constants.

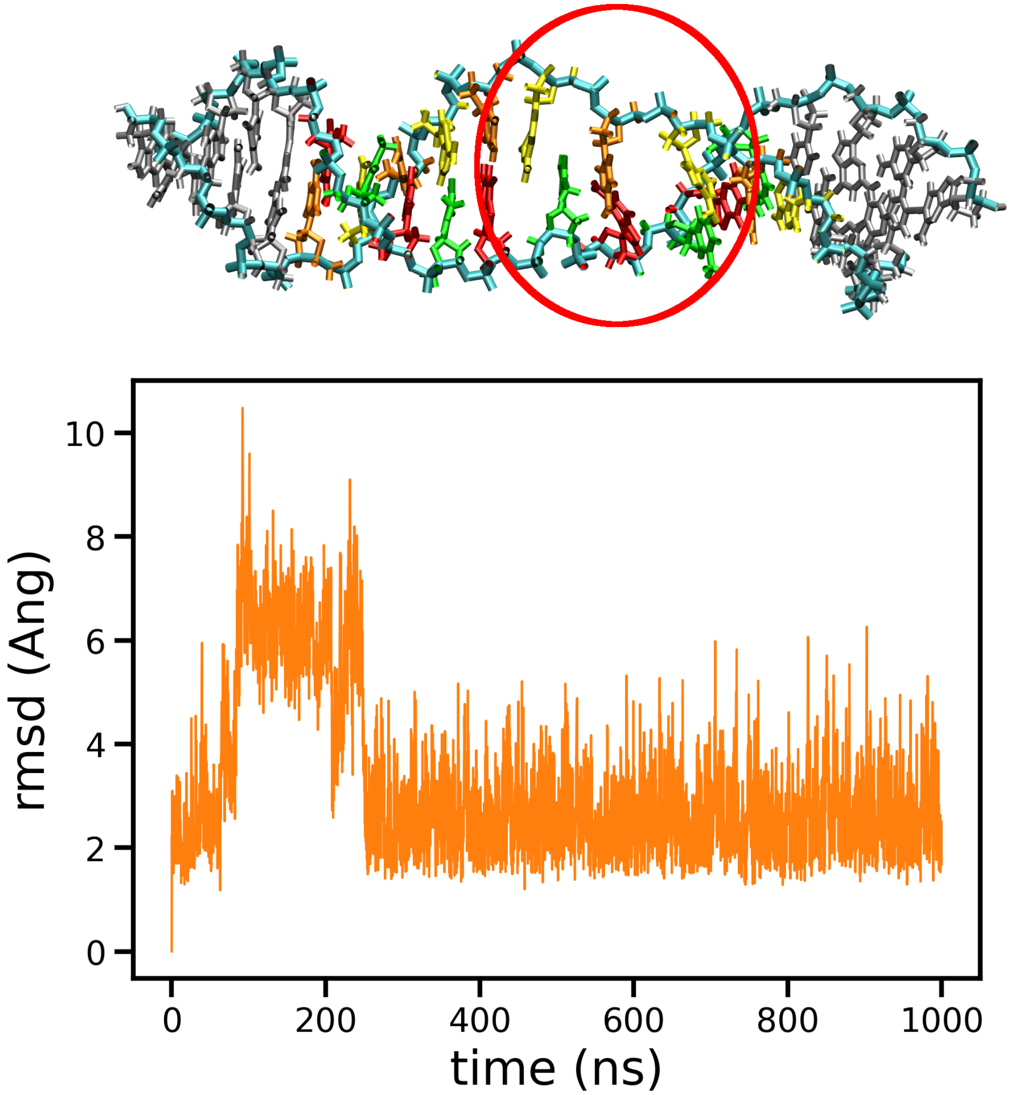

Figure S7: Top: representative snapshot showing the transient departure from B-DNA for simulations of poly-AC with CHARMM36 in the absence of a pulling force and by implementing a strong restraint. In this instance, we observe the presence of local unpairing or unstacking of intact base pairs (red circle). Bottom: time evolution of the rmsd for the same simulation as in the top panel, showing the transient departure from B-DNA between about 100 ns and 200 ns.

7 Anisotropy of persistence length according to global and local definitions

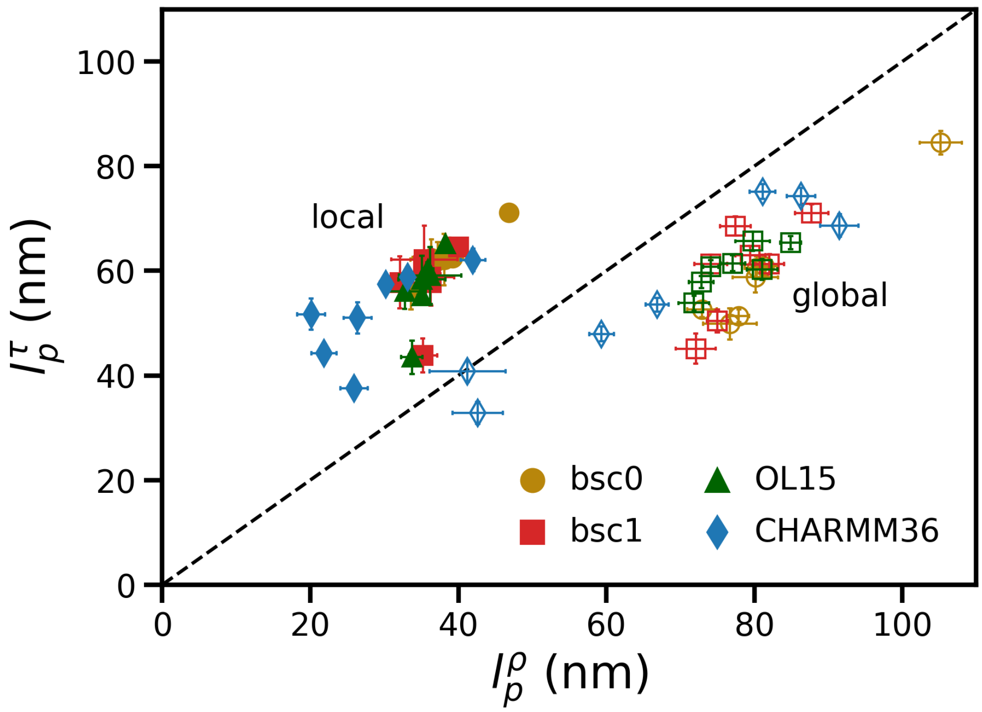

Figure S8: Anisotropy of persistence length, characterized as a scatter plot between roll (l_p^ρ) and tilt (l_p^τ) persistence length. The black line is the bisector of the first quadrant. Empty symbols correspond to the global definition employed in the main text (compare Fig. 7b there) and lie below the bisector, meaning that $l_p^\rho > l_p^\tau$. In contrast, for the local definition (full symbols), the trend is inverted and $l_p^\rho < l_p^\tau$.

References

(S1) C. Maffeo, L. Quednau, J. Wilson and A. Aksimentiev, *Nature Nanotechnology* **18**:238 (2023)
